# [Retracted] Glycyrrhizin protects mice against renal ischemia‑reperfusion injury through inhibition of apoptosis and inflammation by downregulating p38 mitogen‑activated protein kinase signaling

**DOI:** 10.3892/etm.2023.12223

**Published:** 2023-09-25

**Authors:** Shaojun Ye, Yi Zhu, Yingzi Ming, Xingguo She, Hong Liu, Qifa Ye

Exp Ther Med 7:1247–1252, 2014; 10.3892/etm.2014.1570

Following the publication of this paper, it was drawn to the Editors’ attention by a concerned reader that the western blotting data shown in Fig. 2A and 4 were strikingly similar to data appearing in different form in other articles written by different authors at different research institutes that had either already been published elsewhere prior to the submission of this paper to *Experimental and Therapeutic Medicine* or were under consideration for publication at around the same time. In view of the fact that these data had already apparently been published previously, the Editor of *Experimental and Therapeutic Medicine* has decided that this paper should be retracted from the Journal. After having been in contact with the authors, they agreed with the decision to retract the paper. The Editor apologizes to the readership for any inconvenience caused.

